# Buffalo Medical and Surgical Association

**Published:** 1885-01

**Authors:** Frederick Peterson

**Affiliations:** Secretary


### Society Reports.

### Buffalo Medical and Surgical Association.

*Adjourned Meeting Nov. 11, 1884.*

President, F. W. Bartlett, in the Chair.

The minutes of the last meeting were read and approved.

The subjects for the evening being reports of cases and voluntary communications, Dr. Wetmore brought forward a little girl of Irish parentage, aged five years, who had a nævus covering the most of her face from forehead to chin, and upon which there grew an abundance of hair. The nose was flattened, the eyebrows bushy and grown together, and, upon the whole, the child had rather a Simian appearance. The doctor dilated upon the varieties of the nævus vasculosus, the venous, capillary and arterial. This he said was hardly of either class, for here the pigmentation was remarkable, and also the morbid condition of the hair follicles. The mother has had two children since, who are perfect. The mother says the cause of this was a fright by some masked boys in the fifth month of pregnancy. The doctor believed the cause of these malformations to be impressions upon the fœtus in utero by the emotions of the mother. He thought careful dissection might remove much of this deformity, and a cicatrix would be less unsightly.

Dr. Cronyn felt that he must say something about this subject. He had seen the child when smaller. As to the relation between maternal emotions and birth-marks, there was a wide difference of opinion. The coincidences were not sufficient to warrant such a conclusion, scientifically. It is an inexplicable lusus naturæ. As to treatment, dissection of the skin was the only means. The child would never become intelligent, nor would it seem to belong to the human race.

Dr. Hayd mentioned a case where there was a similar nævus upon the back, but having the shape of a mouse. The mother said it was caused by being frightened by a mouse during her pregnancy.

Dr. Hubbell also thought the etiology at present inexplicable. In the treatment by dissections, there will be considerable contraction, and serious deformities may be produced, especially of the eyelids. Transplantation of skin should be added.

Dr. Stockton thought the thanks of the association due to Dr. Wetmore for exhibiting so instructive a case.

Dr. Montgomery also believed transplantation of skin would be necessary in such an operation.

Dr. Bartlett was sure the cicatrization at the completion of the operation would be worse looking than the nævus as it was, and doubted the propriety of an operation.

Dr. Wetmore stated that he would dissect off a very small portion of the skin at the time and scrape off epithelium from the arm to place upon it. He had thought some of using Squibb's cantharidal collodion, and also of several depilatory powders, containing chloride of zinc, etc. He has known these last to be successful. As to causation, he knew that Dr.

Cronyn would oppose the theory of maternal emotion. But many others were as strong in their convictions upon his side as he. Consider the functions of the vaso-motor system, which has charge of the vessels and of secretion. Fright or other strong emotion affected this system in different parts of the body. Churchill mentions the case of a mother, who, after a fit of anger, nursed her child, who thereupon died of convulsions. Here the action of the nervous system must have poisoned the milk. He mentioned cases of the hair's turning white after emotions of grief or fear.

Dr. Cronyn said these cases of coincidence of maternal emotions and birth-marks were very much like the alarm of women approaching the menopause. The millions of women who have no trouble at the climacteric period are never heard from, but the few who do are always in mind. He could see no analogy in the whitening of hair and the appearance of these nævi upon the body. The doctor took exception to the case quoted as from Churchill. The cause of the convulsions was not poisoned milk. There was no milk. The child sucked at the breast in vain, until an excess of gastric juice produced in it gastromalacia. He had known of two such cases in which he had been able to verify his diagnosis by post mortem examination. The vaso-motor influence was sufficient to stop the secretion of milk and turn the hair gray.

Dr. Bartlett asked Dr. Cronyn if any operation upon the jaw, such as the pulling of a tooth, during pregnancy, would produce hare-lip in the child.

Dr. Cronyn answered no. Indeed, if there was severe toothache, he would pull the tooth out to prevent abortion.

Dr. Hubbell stated that in twelve years of practice he had extracted teeth in pregnancy many times without causing harelip in the children.

Dr. Wetmore thought emotions, as a rule, increased secretion, as in hysteria the renal, and did not stop it. What if the milk were already in the breast before the fright?

A member said it would be re-absorbed.

Dr. Coakley asserted that physiology has established the fact that in emotions the secretions are augmented. It was manifestly so with the lacteal under the influence of the emotions of joy, grief, etc. He believed the milk in the tubes could no more be re-absorbed than urine from the bladder. He thought one took a peculiar position who said milk could not produce, in the nursling, great effect, such as diarrhœa, convulsions, etc. But he was sure that maternal emotions did not influence children in utero to cause malformations.

Dr. Cronyn knew that Dr. Coakley could not have got up to refute anything he had said, for he had not done so. He believed, with Dr. Coakley, also, that nerves have a great influence upon the breast.

Dr. Hayd thought Dr. Coakley right as to the breast. He believed the milk could not be re-absorbed.

Dr. Peterson said he could not see the analogy drawn by Dr. Coakley between the mammary gland and the genito-urinary system. Why did the doctor say urine could be absorbed by the bladder as easily as milk by the breast? The urine was not secreted in the bladder, but the epithelium in the whole extent of the tubuli lactiferi was capable of secreting milk. Why could not the fat and serum of milk be re-absorbed by the mammary epithelium, as well as gastric juice by the gastric epithelium or fat by the lacteals?

Dr. Stockton said the point of discussion had been forgotten. It was, can the milk of the mother poison the child? He believed it could not.

Dr. Cronyn again referred to the action of the gastric juice upon the walls of the stomach. He said that it was now an established fact in English law that most sudden deaths of children in bed, hitherto supposed to be due to the overlying of the mother, were really caused by this process of self-digestion in the stomach in gastromalacia.

Dr. Bartlett did not believe in the influence of maternal emotions upon the child in utero in connection with deformities. He mentioned two cases of premature development of teeth in children, with early union of sutures and supernumerary toes and fingers.

Dr. Cronyn spoke of cases of children born with teeth. He knew of one colored child with twelve teeth at birth. All such cases die soon after.

Dr. Wetmore asked: "How about Cæsar?"

Dr. Cronyn said Richard Cœur de Lion was an exception, but he did not remember that Cæsar was.

Dr. Montgomery mentioned the case of a lady, now twenty-four years of age, very frail and delicate, who had all her first teeth before she was eight months old. Her physicians said she would not live after puberty.

Dr. Cronyn spoke of a brother of his, aged sixty years, who had never lost a single tooth in his life.

Dr. Bartlett called attention to thumb-sucking. Recently he visited a lady, who, though twenty-six years of age, could not see him, because she was in the other room sucking her thumb. The thumb had now the appearance of a bird claw.

Dr. Stockton related the history of three generations of hereditary supernumerary toes.

Dr. Hubbell brought up the subject of the new local anæsthetic, muriate of cocaine. He gave the history of its discovery and subsequent uses, as related in recent medical journals. It has its anæsthetic effect upon the eye and mucous membranes, and, in fact, wherever it comes in contact with the nerves. He had used it himself in removing a polypi from the ear, in Bowman's operation, and in removing foreign bodies from the eye, with excellent success. Two drops of a two per cent. solution, repeated at intervals of five minutes, is sufficient. He thought the remedy had a wonderful future.

Dr. Barnes wished to say a few words with regard to osseous tuberculosis, upon which subject Dr. Park had spoken at the last meeting. Dr. Sayre teaches that hip-joint disease is mostly of traumatic origin. Dr. Park had asserted that Dr. Sayre knew nothing about it, but had ridden into fame upon extension and fixation. Dr. Park had stated that American and English surgeons would not accept the tubercular theory because they were obstinate. The speaker believed extension and fixation one of the most important contributions to medical science. We would have to reconcile the cure of hip-joint disease by this means with the idea of tubercular causation.

*Prevailing Diseases*—Dr. Cronyn reported sore throat, diphtheria, scarlet fever, tonsillitis and measles; Dr. Barnes, malarial diseases; Dr. Stockton, remittent fever of a peculiar type; and Dr. Bartlett, dysentery.

The meeting then adjourned.

FREDERICK PETERSON, *Secretary.*

